# Knowledge, Attitude, and Practices of Healthcare Professionals on COVID-19 and Risk Assessment to Prevent the Epidemic Spread: A Multicenter Cross-Sectional Study from Punjab, Pakistan

**DOI:** 10.3390/ijerph17176395

**Published:** 2020-09-02

**Authors:** Usman Rashid Malik, Naveel Atif, Furqan Khurshid Hashmi, Fahad Saleem, Hamid Saeed, Muhammad Islam, Minghuan Jiang, Mingyue Zhao, Caijun Yang, Yu Fang

**Affiliations:** 1 Department of Pharmacy Administration and Clinical Pharmacy, School of Pharmacy, Xi’an Jiaotong University, Xi’an 710061, China; usmanmalik_ucp@hotmail.com (U.R.M.); atifnaveel@stu.xjtu.edu.cn (N.A.); jiangmh2017@xjtu.edu.cn (M.J.); mingyue0204@xjtu.edu.cn (M.Z.); yangcj@mail.xjtu.edu.cn (C.Y.); 2 University College of Pharmacy, University of The Punjab, Lahore, Punjab 54000, Pakistan; furqan.pharmacy@pu.edu.pk (F.K.H.); hamid.pharmacy@pu.edu.pk (H.S.); islam.pharmacy@pu.edu.pk (M.I.); 3 Department of Pharmacy, University of Balochistan, Quetta, Balochistan 87900, Pakistan; fahaduob@gmail.com

**Keywords:** KAP, COVID-19, healthcare professionals, risk assessment, Pakistan

## Abstract

In the current outbreak of novel coronavirus (COVID-19), healthcare professionals (HCPs) have a primary role in combating the epidemic threat. HCPs are at high risk of not only contracting the infection but also spreading it unknowingly. It is of utmost importance to evaluate their knowledge, attitudes, and practices (KAP) and the ability to assess the risks associated with the outbreak. A cross-sectional online survey involving physicians, pharmacists, and nurses was conducted. A 39-itemed questionnaire based on the World Health Organization (WHO)COVID-19 risk assessment tool was shared with healthcare professionals in three purposively selected key divisions of Punjab province. Out of 500 healthcare professionals, 385 responded to the survey. The majority (70%) were aged 22–29 years; 144 (37.4%) physicians, 113 (29.4%) nurses, and 128 (33.2%) pharmacists completed the survey. Overall, 94.8% of healthcare professionals scored adequately (>14) for COVID-19-related knowledge; 97.9% displayed an optimistic attitude (>42) and 94.5% had an adequate practice score (>28). Kruskal–Wallis and Jonckheere–Terpstra tests showed significant differences (*p* < 0.05) in KAP and risk assessment scores among groups; physicians and nurses scored higher as compared to pharmacists. Further research and follow-up investigations on disaster management and risk assessment can help policy-makers better tackle future epidemics.

## 1. Introduction

The first-ever cases of novel coronavirus were reported by the World Health Organization (WHO) regional office in Beijing, China, on 31 December 2019, when a few patients were diagnosed with pneumonia in the city of Wuhan in China [1]. The Wuhan Institute of Virology declared a new strain of coronavirus as a causative agent of this new deadly pneumonia and listed it as a novel coronavirus disease (nCoV-2019) [2]. Later, the disease was officially named as COVID-19 by the WHO [3].

Initially reported in China, the disease started being reported in nearby neighboring countries, and until 20 January 2020, new cases were also confirmed in Thailand, Japan, and South Korea. The first situation report by WHO issued on 21 January 2020 on the novel coronavirus reported 282 confirmed cases of the disease in China and other affected neighboring countries [4]. Since then, the disease transmitted to various parts of the world, and on 30 January 2020, the WHO declared the novel coronavirus disease to be a public health emergency of international concern. Soon after, the disease became a global threat to public health and economies, finally transforming into a pandemic disease. On 11 March 2020, the WHO officially declared COVID-19 as a pandemic disease due to the alarming levels of an upsurge in the spread and severity of the disease [5]. The disease spread rampantly, and according to WHO reports, by 19 July 2020 the disease had shown its notorious presence in around 213 countries with more than 14 million confirmed COVID-19 cases and nearly 0.6 million deaths worldwide [6].

Healthcare workers have a critical role in lowering morbidity and mortality but in doing so they are directly exposed to patients and the causative agents. Preventing nosocomial infections and protecting healthcare workers posed great challenges to the healthcare system during the initial COVID-19 outbreak in China [7]. Healthcare professionals (HCPs) are at a high risk of infection from the patients if they do not have ample knowledge and awareness about the disease or if they do not take adequate precautionary measures. In China, 2050 cases of COVID-19 were reported in healthcare workers as of 20 February 2020; the majority of the cases were due to lower awareness and experience of handling the disease [8]. Avoiding cross-infection from patients along with effective care delivery can be achieved if the healthcare professionals, including physicians, pharmacists, nurses, and other medical staff, have sufficient knowledge, a positive attitude, and better practices about COVID-19. In addition, better preventive policies and risk assessment of healthcare teams are crucial for an effective response to new emerging pandemics such as COVID-19 [9].

In Pakistan, the incidence of coronavirus disease was initially reported on 26 February 2020 in two persons who returned from the epidemic-affected region of Iran [10]. Until 14 March 2020, there were only 31 confirmed COVID-19 cases. However, afterwards, a dramatic escalation was observed, and the number of cases rose stupendously. By 21 June 2020, the confirmed cases in Pakistan had crossed a figure of 174,200 and the new cases were gaining momentum [11]. Deaths of healthcare professionals as a result of exposure to COVID-19 patients have been reported in countries including the USA, the UK, China, and Italy [12,13]. A recent study reported the deficiencies in the awareness and preparedness of medical professionals regarding COVID-19 in Pakistan and demonstrated that frontline health workers were not well-prepared to prevent and control the infection [14]. Keeping in view the severity of the outbreak and the importance of healthcare professionals working with scarce resources to combat COVID-19, it was pertinent to evaluate their knowledge, attitude, practices, and risk assessment skills.

## 2. Methodology

### 2.1. Study Design

A cross-sectional study to examine the knowledge, attitude, practices, and risk assessment of HCPs regarding coronavirus and its associated disease (COVID-19) was conducted. These data were collected from 21 March 2020 after the COVID-19 cases began increasing in Pakistan, and were completed on 20 April 2020.

### 2.2. Study Setting

**Figure 1:**
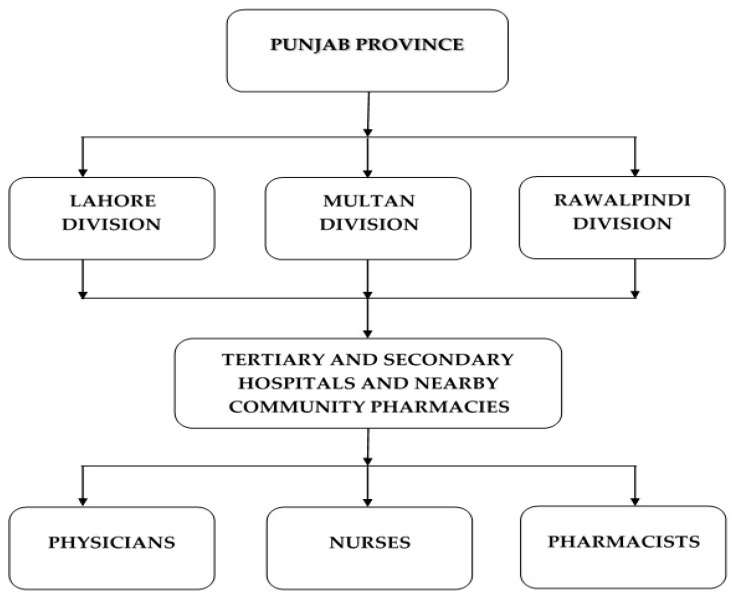
Study Population.

Pakistan is composed of five provinces, with Punjab being the most populous province of Pakistan (hosting >50% of total population) having a population of about 110 million [15]. Out of nine administrative divisions in the Punjab province, three key divisions (Lahore, Multan, and Rawalpindi, representing the north-east, southern, and northern parts of Punjab province, respectively) were purposively selected. Healthcare professionals working in tertiary and secondary hospitals along with nearby located community pharmacies were chosen as the target population, thus covering both public and private sector HCPs (Figure 1).

### 2.3. Study Participants

The study was conducted on doctors (general physicians), hospital and community pharmacists, and nurses who dealt with management and control of the COVID-19 disease. These participants included HCPs working in various private and public sector (secondary and tertiary care) hospitals and those working at community pharmacies located close to the hospitals. All registered HCPs with their respective councils (Pakistan Medical and Dental Council, Pakistan Nursing Council, and Pharmacy Council of Pakistan) who were dealing with COVID-19 cases, regardless of their experience, age, gender, and socioeconomic status, were included in the study. Unregistered HCPs and those returning incomplete surveys were excluded during the data analysis phase. Participation in the survey was purely on a voluntary basis, and the participants could choose to quit the survey at any stage. In order to obtain robust and complete information, we requested that the participants responded to all questions of the survey.

### 2.4. Sampling Strategy

There are 107,112 physicians (including the general physicians and specialists) [16], 64,846 nurses [17], and 21,954 pharmacists in Punjab province, thus totaling to a population size of 193,912 [18]. Raosoft sample size calculator was used to select the sample size with a 95% confidence level, 5% margin of error, and a population size of 193,912. The resultant sample size of 384 was calculated to be a representative of the health care professional population in Punjab province. Keeping in mind a 20% dropout, in order to achieve an optimal response rate, a total of 500 healthcare professionals were approached online through the WhatsApp© application (Copyright 2020 WhatsApp Inc., Menlo Park, CA, USA) and cellular phone calls by using the purposive sampling method. 

### 2.5. Study Instrument

The questionnaire was designed based on the latest WHO risk assessment and management of healthcare workers in the context of COVID-19 tool [19] and published literature on previously spread viral epidemics in various parts of the world [20,21]. Initially, the questionnaire consisted of 60 questions on knowledge, attitude, practices, and risk assessment. However, after content and face validity by five experts in clinical practice, pharmacy, and academia (two classified physicians, two pharmacists, and one professor of pharmacy practice), the questionnaire was reduced to 39 questions/items (See Supplementary file). Initially, a pilot study was conducted and data of 50 participants were used to determine the internal consistency of the questionnaire by calculating the Cronbach’s alpha value. The Cronbach’s alpha value of the questionnaire was calculated to be 0.74, which indicated an acceptable level of internal consistency.

The final questionnaire had four sections with 39 questions/items. The basic information from all the participants regarding their age, gender, marital status, level of education, occupation, and type of organization was obtained in the demographics section. The second section contained 19 questions about the basic knowledge of coronavirus disease with three options (“yes”, “no”, and “do not know”). The knowledge was assessed by giving the value 1 to a correct answer and 0 to the wrong answer. The “do not know” response was also processed as 0. The scale measured knowledge score from a maximum of 19 to minimum of 0. Scores <9 were taken as poor, 9–14 average, and >14 as adequate knowledge of COVID-19. The third section constituted 12 questions on the attitude of healthcare professionals and was rated using a 5-point Likert scale varying from “strongly disagree” to “strongly agree”. The attitude was assessed by giving value 1 to “strongly disagree” and 5 to the “strongly agree” response. Reversed scoring was utilized for negative questions. The scale measuring attitudes ranged from 12 to 60. Scores ≤42 were taken as negative while >42 were taken as a positive attitude towards COVID-19. The fourth section had 8 questions on COVID-19-related practices and risk assessment (4 on practices and 4 on risk assessment) rated on a 5-point Likert scale: “always”, “mostly”, “sometimes”, “rarely”, and “never”. This scale measured practices and risk assessment from a maximum score of 40 to a minimum of 8. Scores ≤28 were taken as poor practices while >28 were taken as good practices towards COVID-19.

### 2.6. Data Collection

Keeping in mind the lockdown situation, for the purpose of study, we developed an online Google form of the questionnaire that we shared with healthcare professionals. The Google form was shared with the participants through the WhatsApp platform and the participants were requested via cellular phone calls to fill the online survey. Continuous weekly reminders were given both through cellular phone calls and WhatsApp to ensure optimal participation. Participants were also guided about the aims and objectives of the study and informed consent was obtained verbally to make sure they understand each aspect of the study before filling the survey form.

### 2.7. Ethical Approval

Ethical approval was obtained from the Human Ethics Committee at the University College of Pharmacy, University of the Punjab, Lahore, Pakistan, no. D/HEC/112/UCP2340 and the Biomedical Ethics Committee at Xi’an Jiaotong University, Xi’an, China.

### 2.8. Statistical Analysis

The data from the Google forms was imported to SPSS (version 22.0, IBM, Chicago, IL, USA) and analyzed. Descriptive statistics were performed by calculating the frequencies and proportions. The scores on knowledge, attitude, and practices & risk assessment were calculated from a total of 19, 60, and 40 points, respectively. The correct responses for all the questions were determined from the guidelines developed by the WHO for general public and healthcare workers. Data were checked for normality by the Kolmogorov–Smirnov test and Shapiro–Wilk’s test. These tests indicated that the data were not normally distributed, with a *p*-value < 0.05 and skewness of −0.702(Standard Error (S.E.) = 0.124), −0.317 (S.E. = 0.124), and −1.405 (S.E. = 0.124) for scores on knowledge, attitude, and practices, respectively. The Kruskal–Wallis test was used to evaluate the significant differences among physicians, pharmacists, and nurses and their scores on knowledge, attitude, practices, and risk assessment. The Jonckheere–Terpstra test was used to confirm the trend of association.

## 3. Results

### 3.1. Demographics of Participants

Out of a total 500 healthcare professionals approached, 385 completed the online survey. The response rate of the survey was 77%. The ages of health care professionals ranged from 22 to 68 years, with a mean age of 28.73 ± 6.31 years. A majority of the participants (69.4%) belonged to the age range of 22–29 years. Out of the 385, 144 (37.4%) were physicians, 113 (29.4%) were nurses, and 128 (33.2%) were pharmacists. Overall, 53% of all the participants were females. Around 51% of participants were from the public sector while 49% came from the private sector. A majority of the participants (88%) belonged to urban regions of Punjab province. Table 1 shows the baseline characteristics of the respondents.

### 3.2. Knowledge of Healthcare Professionals

Among the 385 participants, 94.8% partakers scored >14, and the average knowledge score of participants was 17.31 ± 1.40. In the current cohort, all of the participants were aware that the disease was a viral infection and were also familiar with the most commonly observed symptoms of COVID-19. Among them, 99.0% knew that the disease could be transmitted through infected humans and animals; 80% knew that the COVID-19 virus is a virus that is related to the severe acute respiratory syndrome coronavirus-2 (SARS-CoV) and Middle East respiratory syndrome coronavirus-2 (MERS-CoV) family; and 95.8% were aware of the asymptomatic presence of COVID-19 in people who recently visited virus-affected areas, with this being a potential source of disease spread. However, 42.1% believed that the disease could be transmitted through contaminated food and 17.9% believed that COVID-19 was similar to the normal flu or cold. Almost, 37% of healthcare workers believed that antibiotics could be useful in the treatment of COVID-19 (See Table 2).

About 96.0% were aware of the fact that virus could survive on different objects such as doors, windows, beds, and tables; 99.7% knew that isolation of the infected patients is a necessity to avoid or prevent disease transfer to other people; and 97.4% knew that the incubation period for symptoms to appear ranges from 1 to 2 weeks. About 97% knew that patients with comorbidities are at a higher risk of getting infected, and 99.7% knew that immune-compromised, old age people, and healthcare professionals working closely with infected people were at increased risk of infection. A total of 84.7% of healthcare professionals believed that they were well prepared to deal with COVID-19 in the case of an outbreak in the country (See Table 2).

### 3.3. Attitude of Healthcare Professionals

Overall, 97.9% participants scored >42, showing a positive attitude, with a mean score of 50.69 ± 3.96. The majority of the participants (>97%) agreed that the disease could be transmitted by coughing and sneezing and that regular hand washing and the use of sanitizer would help prevent the spread of infection. Moreover, around 94% agreed that wearing masks can help prevent COVID-19 transmission to other people and 97.6% agreed that isolating infected patients could be beneficial in reducing the risk of cross-infection. More than 98.0% agreed that avoiding frequent touching of the nose, mouth, and eyes could reduce the risk of infection, and 92.2% of participants also agreed that avoiding contact with doors, furniture, and other objects significantly reduce the risk of infection. Out of the remaining 7.8%, only 1.1% disagreed on the possibility of transmission through objects, while 6.7% stayed neutral (See Table 3).

More than 92.0% of subjects also expressed that if a vaccine is developed against COVID-19, it can significantly prevent the epidemic spread. However, a mixed response was observed for the use of antibiotics in the prevention of the infection. About 39.0% of healthcare professionals agreed antibiotics could be useful in preventing the COVID-19 infection, while 7.5% remained neutral and 53.5% disagreed with the statement (See Table 3).

### 3.4. Practices and Risk Assessment of Healthcare Professionals

About 94.5% of healthcare professionals scored >28 and showed better practices towards disease management with a mean score of 35.97 ± 4.15. About 93.0% of participants almost always advised their patients to eat properly cooked food and 96.9% advised using soaps and sanitizer for regular hand and face washing. Moreover, more than 93% of healthcare professionals in most interactions with people advise them to keep themselves warm and hydrated, and 89% advise avoiding close contact with people with cough and flu-like symptoms. Out of remaining population, 5.5% sometimes advised and 2.9% rarely advised patients to avoid close contact with people with flu and cold-like symptoms.

The risk assessment revealed that more than 92.0% of healthcare workers almost always preferred to use personal protective equipment (PPE) during interaction with COVID-19-suspected patients. More than 91% almost always perform a hand hygiene and washing procedure before or after any medical intervention or procedures. About 89% of participants, in a majority of interactions, perform hand hygiene after touching a patients’ surroundings such as beds, doors, tables, and almost 86% of healthcare staff observe social distancing in a majority of interactions and avoid unnecessary contact with the patients. From the remaining 14.0%, 7.0% sometimes avoid unnecessary contact while the other 7.0% rarely or never observe social distancing as it is difficult for them to do so because of the continuous exposure to patients (See Table 4).

**Figure 2:**
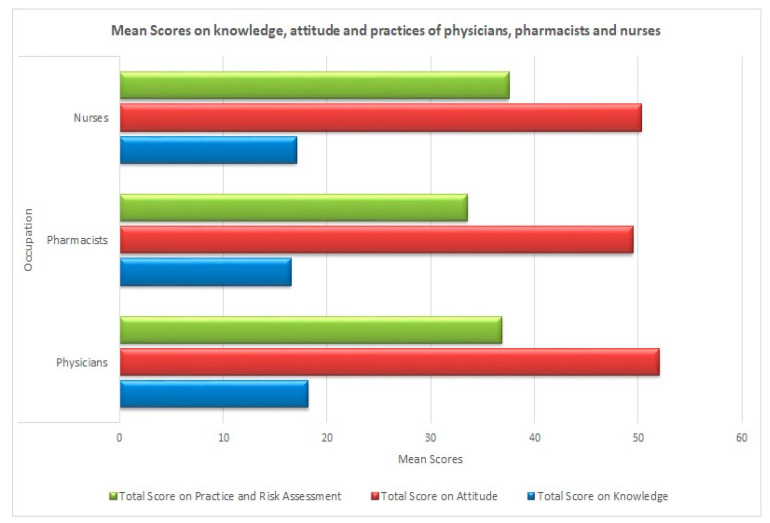
Mean scores on knowledge, attitude and practices of physicians, pharmacist and nurses.

The Kruskal–Wallis test demonstrated significant differences (*p*-value < 0.05) in the scores on knowledge, attitude, practices, and risk assessment of different healthcare professionals (Table 5). Overall, the physicians achieved significantly higher scores on knowledge and attitude as compared to pharmacists and nurses. No significant difference was observed on knowledge and attitude scores between pharmacists and nurses. However, the scores on practices and risk assessment demonstrated that the nurses and physicians scored significantly higher as compared to pharmacists (See Figure 2). 

The Jonckheere–Terpstra test confirmed that knowledge and attitude towards COVID-19 were significantly associated (*p* < 0.001) with occupation. A positive trend was further reported, whereby physicians had more knowledge and carried a positive attitude towards COVID-19 as compared to nurses and pharmacists (τ = 0.411 and 0.398, respectively) (See Table 6).

## 4. Discussion

This study exclusively targeted the healthcare staff that would be directly or indirectly in contact with suspected or confirmed COVID-19 patients. In current study, more than 94% of HCPs had adequate knowledge about COVID-19, which is relatively better than earlier reported studies conducted in other countries including Egypt, Iran, and Greece [22,23,24].

The clinical symptoms most commonly observed in COVID-19 patients in a recent study were fever, cough, fatigue, or myalgia and dyspnea [25,26]. All of the respondents were well aware of most common symptoms, which showed a considerate level of understanding. Moreover, the awareness about the effectiveness of hygienic principles such as regular hand washing, sanitizer usage, isolation of patients, and self-confinement at homes for the prevention of COVID-19 is a positive sign. The basic health measures such as washing hands regularly, staying at home, maintaining social distancing, and covering the mouth and nose during coughing and sneezing were effective in controlling and preventing a previously unfolded SARS epidemic in China [27]. Moreover, these measures have proved to be effective in preventing COVID-19 virus transmission [28]. Approximately 90% of participants in our study exhibited better practices of hygiene and handwashing before and after interacting with the patients, which is quite high as compared to the findings of a study conducted in Greece where only 1 in 4 HCPs had a hand-washing routine before and after the patient interaction [23].

Dissimilarity was observed between the knowledge, attitude, and practices of healthcare professionals. Physicians and nurses, in particular, had significantly higher scores as compared to pharmacists, reflecting a need for improvements in terms of practices and disaster responsiveness. This kind of variation among healthcare workers was also reported in Saudi Arabia after the outbreak of MERS disease in 2016 [20]. However, our findings with pharmacists lacking in COVID-19-related knowledge and apt practices were contrary to a recent survey reported from Pakistan in which the pharmacists demonstrated better practices as compared to other healthcare colleagues [29].

The respondents’ mixed responses about the risk of transmission of the disease through virus-contaminated food and the beneficial and effective role of antibiotics for the treatments of COVID-19 reflects uncertainty and misperception among healthcare workers. Analytically speaking, until now, no evidence has yet reported the risk of transfer of COVID-19 virus to healthy people through contaminated food. The foodborne gastrointestinal viruses often lead to transmission of the virus through food, but COVID-19 mainly transmits from person to person, and transmission through food has not been reported from any part of the world [30,31].

A few antiviral, antimalarial, and anti-inflammatory drugs have shown some benefits in terms of therapy. However, the usefulness of antibiotics as a therapy for COVID-19 is still unclear. The footprints of ambiguity about antibiotics were clearly visible in our study, however, a high number of HCPs (60.5%) believed that antibiotics are not useful therapy for COVID-19, a reasonably high percentage as compared to findings from a study in Egypt where only 38% of participants believed so [22]. A few recent studies suggested a supportive and symptomatic treatment approach for disease management and treating secondary bacterial infections [26,32]. However, antibiotics do not work against any viruses and are only recommended against bacterial infections arising from COVID-19 and not as a preventive measure or a treatment for coronavirus infection [33]. Following the rumors about the possible use of azithromycin in combination with hydroxychloroquine, the already existing debate about the role of antibiotics in COVID-19 escalated [34]. A randomized controlled trial conducted recently has shown that the use of azithromycin in combination with hydroxychloroquine may be effective in eliminating COVID-19. However, the trial involved fewer patients [35] and warrants further research to collect evidence on the effectiveness of azithromycin and/or hydroxychloroquine to treat/prevent a viral disease [36].

## 5. Limitations

The current study had certain limitations. The study was conducted in three key divisions of one province and thus the results may not be generalizable to the rest of country. Secondly, most of the healthcare professionals came from urban areas. Rural areas were not easily accessible during the pandemic outbreak, and therefore not enough responses were achieved from rural parts of Punjab. Thirdly, healthcare staff other than physicians, nurses, and pharmacists were not involved in the study and their practices went unreported. Fourth, the survey was conducted online using WhatsApp and, therefore, many of the HCPs were not able to be contacted for participation. Fifth, the HCPs were inquired about their earlier experiences with COVID-19 patients, which may have led to recall bias. Finally, owing to the exploratory nature of the study, the inherent selection bias cannot be overruled. Participants’ age may also be one of the potential confounding factors.

## 6. Conclusions

The study revealed that most of the participants were well primed to deal with the pandemic. Pharmacists exhibited relatively lower levels of knowledge and their practices indicated that they were at a higher risk of contracting infections as compared to physicians and nurses. Interestingly, due to a lack of evidence, the healthcare professionals were not certain about use of antibiotics to treat or prevent COVID-19. It is suggested that the government should take necessary measures to train all healthcare stakeholders for the emergency preparedness and any other environmental or health-related calamity. Further research and follow-up investigations are needed to evaluate the readiness of HCPs in terms of disaster management and risk assessment to avert future public health crises.

## Figures and Tables

**Table 1:** Demographic characteristics of study respondents (*N* = 385).

Characteristics	Frequency (*n*)	Percentage (%)
Age Groups (Mean Age = 28.73 ± 6.31 years)		
<=25	142	36.9
26–29	125	32.5
30+	118	30.6
Gender		
Male	181	47.0
Female	204	53.0
Marital Status		
Married	195	50.6
Unmarried	190	49.4
Highest Level of Education		
Bachelor	181	47.0
Masters	170	44.2
Doctorate	34	8.8
Occupation		
Physician	144	37.4
Pharmacist	128	33.2
Nurse	113	29.4
Workplace Organization Type		
Public	196	50.9
Private	189	49.1
Locality		
Urban	340	88.3
Rural	45	11.7

**Table 2:** Responses to COVID-19 knowledge items.

Items	Yes *n* (%)	No *n* (%)	Do Not Know *n* (%)
Have you heard about the novel corona virus and the related terms COVID-19 or 2019-nCoV?	385 (100%)	0 (0%)	0 (0%)
COVID-19 disease is a viral infection.	385 (100%)	0 (0%)	0 (0%)
COVID-19 can be transmitted through close contact with infected people and infected animals.	381 (99%)	4 (1%)	0 (0%)
COVID-19 virus can be transmitted through contaminated food and water.	162 (42.1%)	198 (51.4%)	25 (6.5%)
Fever, sore throat, cough, and shortness of breath are possible symptoms of COVID-19 infection.	385 (100%)	0 (0%)	0 (0%)
The novel coronavirus is a similar virus as SARS-CoV and MERS-CoV.	308 (80.0%)	31 (8.1%)	46 (11.9%)
Does the virus survive on surfaces of doors, tables and other objects?	371 (96.4%)	9 (2.3%)	5 (1.3%)
Is the COVID-19 infection the same illness as flu or cold?	69 (17.9%)	309 (80.3%)	7 (1.8%)
Is there any laboratory test to confirm the presence of COVID-19 infection?	369 (95.8%)	12 (3.1%)	4 (1.0%)
The incubation period of COVID-19 infection is 1–2 weeks.	375 (97.4%)	3 (0.8%)	7 (1.8%)
Can COVID-19 infection be caught from a person who presents no symptoms and has recently visited the affected area?	369 (95.8%)	10 (2.6%)	6 (1.6%)
A vaccine for the COVID-19 virus is now available in the market.	14 (3.6%)	361 (93.8%)	10 (2.6%)
Antibiotics are useful for the treatment of COVID-19 infection.	142 (36.9%)	233 (60.5%)	10 (2.6%)
People with a compromised immune system and old age people are at more risk of developing the infection.	384 (99.7%)	1 (0.3%)	0 (0%)
Patients with comorbidities are at more risk of developing the infection.	373 (96.9%)	7 (1.8%)	5 (1.3%)
Health care workers and hospitalized patients who are near to infected patients are at more risk of developing the infection.	384 (99.7%)	1 (0.3%)	0 (0%)
People in crowded places are at increased risk of getting affected by the disease.	385 (100%)	0 (0%)	0 (0%)
Patients of COVID-19 infection should be immediately isolated to avoid the transfer of infection to other people.	384 (99.7%)	1 (0.3%)	0 (0%)
Healthcare professionals are well prepared to cater to the people in case there is a spread of COVID-19 disease.	326 (84.7%)	50 (13.0%)	9 (2.3%)

COVID-19 = Coronavirus disease 2019, 2019-nCoV = Novel coronavirus disease 2019, SARS-CoV = Severe acute respiratory syndrome coronavirus and MERS-CoV = Middle-East respiratory syndrome coronavirus.

**Table 3:** Responses to COVID-19-related attitude items.

Items	SD *n* (%)	D *n* (%)	N *n* (%)	A *n* (%)	SA *n* (%)
The disease can be transmitted by coughing and sneezing.	4 (1.0%)	2 (0.5%)	3 (0.8%)	106 (27.5%)	270 (70.1%)
Transmission of COVID-19 infection can be prevented through wearing masks.	0 (0%)	6 (1.6%)	18 (4.7%)	182 (47.3%)	179 (46.5%)
Transmission of COVID-19 infection can be prevented through washing hands and face regularly with antiseptics and sanitizers.	2 (0.5%)	0 (0%)	8 (2.1%)	146 (37.9%)	229 (59.5%)
Transmission of COVID-19 infection can be prevented through the isolation of COVID-19-infected patients.	0 (0%)	0 (0%)	9 (2.3%)	141 (36.6%)	235 (61.0%)
Transmission of COVID-19 infection can be prevented by taking antibiotics.	114 (29.6%)	92 (23.9%)	29 (7.5%)	64 (16.6%)	86 (22.3%)
Restricting the travel of COVID-19-infected people to other areas of the world and of people in other areas to affected areas can be beneficial to prevent the spread of the infection.	1 (0.3%)	1 (0.3%)	12 (3.1%)	158 (41.0%)	213 (55.3%)
Having a healthy and well-cooked diet can be helpful in reducing the risk of getting the novel coronavirus disease.	1 (0.3%)	13 (3.4%)	36 (9.4%)	189 (49.1%)	146 (37.9%)
Avoiding touching the nose, mouth, and eyes can reduce the risk of infection.	0 (0%)	0 (0%)	7 (1.8%)	180 (46.8%)	198 (51.4%)
Avoiding touching the surface of doors, furniture, or other things can be helpful in preventing the disease.	1 (0.3%)	3 (0.8%)	26 (6.8%)	195 (50.6%)	160 (41.6%)
If a vaccine is developed against the novel coronavirus, it can significantly reduce the epidemic spread.	1 (0.3%)	2 (0.5%)	26 (6.8%)	183 (47.5%)	173 (44.9%)
The available information about COVID-19 disease is sufficient in Pakistani society.	18 (4.7%)	58 (15.1%)	43 (11.2%)	138 (35.8%)	128 (33.2%)
The government in our country has all the necessary healthcare facilities and is able to control the epidemic situation.	35 (9.1%)	63 (16.4%)	36 (9.4%)	138 (35.8%)	113 (29.4%)

COVID-19 = Coronavirus disease 2019, SD = strongly disagree, D = disagree, N = neutral, A = agree and SA = strongly agree.

**Table 4:** COVID-19 related practices and risk assessment.

Items	Always *n* (%)	Mostly *n* (%)	Sometimes *n* (%)	Rarely *n* (%)	Never *n* (%)
I advise the general public to eat thoroughly cooked food, especially meat products.	247 (64.2%)	111 (28.8%)	21 (5.5%)	5 (1.3%)	1 (0.3%)
I advise the public to keep themselves warm and hydrated.	207 (53.8%)	152 (39.5%)	23 (6.0%)	2 (0.5%)	1 (0.3%)
I advise people to use soap or sanitizer regularly to wash their hands and face.	276 (71.7%)	97 (25.2%)	11 (2.9%)	1 (0.3%)	0 (0%)
I advise the general public to avoid close contact with people with cough and flu-like symptoms.	234 (60.8%)	111 (28.8%)	21 (5.5%)	11 (2.9%)	8 (2.1%)
During interaction with a COVID-19 patient, I wear the necessary personal protective equipment (PPE) such as masks, gloves, gown, etc.	238 (61.8%)	117 (30.4%)	24 (6.2%)	4 (1.0%)	2 (0.5%)
I perform hand hygiene before and after touching COVID-19 patients or before and after performing an aseptic procedure.	252 (65.5%)	101 (26.2%)	24 (6.2%)	6 (1.6%)	2 (0.5%)
I perform hand hygiene after touching a patient’s surroundings such as beds, tables, doors, etc.	219 (56.9%)	125 (32.5%)	28 (7.3%)	9 (2.3%)	4 (1.0%)
I avoid unnecessary close contact, practice social distancing, and keep at least 1 meter distance from patients and other healthcare workers.	233 (60.5%)	98 (25.5%)	27 (7.0%)	13 (3.4%)	14 (3.6%)

COVID-19 = Coronavirus disease 2019.

**Table 5:** Intergroup analysis (Kruskal–Wallis independent sample test).

Characteristics	Study Group	*p*-Value
Total Score on Knowledge	Pharmacist–Nurse	0.162
	Pharmacist–Physician	0.000 *
	Nurse–Physician	0.000 *
Total Score on Attitude	Pharmacist–Nurse	0.310
	Pharmacist–Physician	0.000 *
	Nurse–Physician	0.001 *
Total Score on Practices and Risk Assessment	Pharmacist–Nurse	0.000 *
	Pharmacist–Physician	0.000 *
	Nurse–Physician	0.092

* Significance level (*p*-value < 0.05).

**Table 6:** Intergroup analysis. (Jonckheere–Terpstra trend test)

Study Group	*p*-Values *		
	Knowledge	Attitude	Practice
Physicians + Nurses + Pharmacists	<0.001	<0.001	0.221

* Significance level (*p*-value < 0.05).

## Supplementary Materials

The following are available online at https://www.mdpi.com/1660-4601/17/17/6395/s1, Questionnaire, Table S1: Section II—Knowledge, Table S2: Section III—Attitudes, Table S3: Section IV—Practices and Risk Assessment.

## References

[1] Paules C.I., Marston H.D., Fauci A.S. (2020). Coronavirus Infections—More Than Just the Common Cold. JAMA.

[2] Zhou P., Yang X.-L., Wang X.-G., Hu B., Zhang L., Zhang W., Si H.-R., Zhu Y., Li B., Huang C.-L. (2020). A pneumonia outbreak associated with a new coronavirus of probable bat origin. Nature.

[3] World Health Organization (WHO) Naming the Coronavirus Disease. https://www.who.int/emergencies/diseases/novel-coronavirus-2019/technical-guidance/naming-the-coronavirus-disease-(covid-2019)-and-the-virus-that-causes-it.

[4] World Health Organization (WHO) (2020). Novel Coronavirus Situation Report-1. https://www.who.int/emergencies/diseases/novel-coronavirus-2019/situation-reports.

[5] WHO Director General Media Briefing 11 March 2020 Covid-19 a Pandemic. https://www.who.int/dg/speeches/detail/who-director-general-s-opening-remarks-at-the-media-briefing-on-covid-19—11-march-2020.

[6] World Health Organization (WHO) (2020). Novel Coronavirus Situation Report-181. https://www.who.int/emergencies/diseases/novel-coronavirus-2019/situation-reports.

[7] Wang D., Hu B., Hu C., Zhu F., Liu X., Zhang J., Wang B., Xiang H., Cheng Z., Xiong Y. (2020). Clinical Characteristics of 138 Hospitalized Patients With 2019 Novel Coronavirus—Infected Pneumonia in Wuhan, China. JAMA.

[8] World Health Organization (WHO) Report of the WHO-China Joint Mission on Coronavirus Disease 2019 (COVID-19). https://www.who.int/publications-detail/report-of-the-who-china-joint-mission-on-coronavirus-disease-2019-(covid-19).

[9] Wu Z., McGoogan J.M. (2020). Characteristics of and Important Lessons From the Coronavirus Disease 2019 (COVID-19) Outbreak in China. Summary of a Report of 72314 Cases From the Chinese Center for Disease Control and Prevention. JAMA.

[10] Coronavirus Cases in Pakistan. https://www.pakistantoday.com.pk/2020/02/26/sindh-health-two-coronavirus-cases-confirmed-in-pakistan-confirms-first-coronavirus-case-in-karachi/.

[11] Ministry of National Health Services Regulation and Coordination, G.O.P. COVID-19 Pakistan. http://covid.gov.pk.

[12] Zhan M., Qin Y., Xue X., Zhu S. (2020). Death from Covid-19 of 23 Health Care Workers in China. N. Engl. J. Med..

[13] Anelli F., Leoni G., Monaco R., Nume C., Rossi R.C., Marinoni G., Spata G., De Giorgi D., Peccarisi L., Miani A. (2020). Italian doctors call for protecting healthcare workers and boosting community surveillance during covid-19 outbreak. BMJ.

[14] Khan S., Khan M., Maqsood K., Hussain T., Huda N.-U., Zeeshan M. (2020). Is Pakistan prepared for the COVID-19 epidemic? A questionnaire-based survey. J. Med. Virol..

[15] Bureau of Statistics, Government of Pakistan Census 2017. http://www.pbs.gov.pk/content/provisional-summary-results-6th-population-and-housing-census-2017-0.

[16] Pakistan Medical and Dental Council Registered Physicians in Punjab, Pakistan. http://www.pmdc.org.pk/Statistics/tabid/103/Default.aspx.

[17] Punjab Public Health Agency Nurses and Midwifery in Punjab, Pakistan. http://www.ppha.punjab.gov.pk/news-and-highlights/nursing-and-midwifery.html.

[18] Punjab Pharmacy Council Registered Pharmacists in Punjab, Pakistan. https://punjabpharmacycouncil.com/index_files/Page528.htm.

[19] World Health Organization (WHO) (2020). Risk Assessment and Management of Exposure of Health Care Workers in the Context of COVID-19. https://www.who.int/publications/m/item/risk-assessment-and-management-of-exposure-of-health-care-workers-in-the-context-of-covid-19-data-template.

[20] Asaad A.M., El-Sokkary R.H., Alzamanan M.A., El-Shafei M. (2019). Knowledge and attitudes towards Middle East respiratory syndrome-coronavirus (MERS-CoV) among health care workers in south-western Saudi Arabia. East. Mediterr. Health J..

[21] Al-Hazmi A., Gosadi I., Somily A., Alsubaie S., Bin Saeed A. (2016). Knowledge, attitude and practice of secondary schools and university students toward Middle East Respiratory Syndrome epidemic in Saudi Arabia: A cross-sectional study. Saudi J. Boil. Sci..

[22] Abdel-Wahed W.Y., Hefzy E.M., Ahmed M.I., Hamed N.S. (2020). Assessment of Knowledge, Attitudes, and Perception of Health Care Workers Regarding COVID-19, A Cross-Sectional Study from Egypt. J. Commun. Health.

[23] Papagiannis D., Malli F., Raptis D.G., Papathanasiou I.V., Fradelos E.C., Daniil Z., Rachiotis G., Gourgoulianis K. (2020). Assessment of Knowledge, Attitudes, and Practices towards New Coronavirus (SARS-CoV-2) of Health Care Professionals in Greece before the Outbreak Period. Int. J. Environ. Res. Public Health.

[24] Taghrir M.H., Borazjani R., Shiraly R. (2020). COVID-19 and Iranian Medical Students; A Survey on Their Related-Knowledge, Preventive Behaviors and Risk Perception. Arch. Iran. Med..

[25] Del Rio C., Malani P.N. (2020). COVID-19—New Insights on a Rapidly Changing Epidemic. JAMA.

[26] Huang C., Wang Y., Li X., Ren L., Zhao J., Hu Y., Zhang L., Fan G., Xu J., Gu X. (2020). Clinical features of patients infected with 2019 novel coronavirus in Wuhan, China. Lancet.

[27] Del Rio C., Malani P.N. (2020). 2019 Novel Coronavirus—Important Information for Clinicians. JAMA.

[28] Mitjà O., Clotet B. (2020). Use of antiviral drugs to reduce COVID-19 transmission. Lancet Glob. Health.

[29] Saqlain M., Munir M.M., Rehman S.U., Gulzar A., Naz S., Ahmed Z., Tahir A.H., Mashhood M. (2020). Knowledge, attitude, practice and perceived barriers among healthcare workers regarding COVID-19: A cross-sectional survey from Pakistan. J. Hosp. Infect..

[30] USA Food and Drug Administration Food Safety and Coronavirus Disease 2019. https://www.fda.gov/food/food-safety-during-emergencies/food-safety-and-coronavirus-disease-2019-covid-19.

[31] World Health Organization (WHO) (2020). COVID-19 and Food Safety: Guidance for Food Businesses.

[32] Jiang F., Deng L., Zhang L., Cai Y., Cheung C.W., Xia Z. (2020). Review of the Clinical Characteristics of Coronavirus Disease 2019 (COVID-19). J. Gen. Int. Med..

[33] World Health Organization (WHO) Coronavirus Disease 2019, Advice for Public. https://www.who.int/emergencies/diseases/novel-coronavirus-2019/advice-for-public/myth-busters.

[34] Million M., Lagier J.-C., Gautret P., Colson P., Fournier P.-E., Amrane S., Hocquart M., Mailhe M., Esteves-Vieira V., Doudier B. (2020). Early treatment of COVID-19 patients with hydroxychloroquine and azithromycin: A retrospective analysis of 1061 cases in Marseille, France. Travel Med. Infect. Dis..

[35] Gautret P., Lagier J.-C., Parola P., Hoang V.T., Meddeb L., Mailhe M., Doudier B., Courjon J., Giordanengo V., Vieira V.E. (2020). Hydroxychloroquine and azithromycin as a treatment of COVID-19: Results of an open-label non-randomized clinical trial. Int. J. Antimicrob. Agents.

[36] Molina J.-M., Delaugerre C., Le Goff J., Mela-Lima B., Ponscarme D., Goldwirt L., De Castro N. (2020). No evidence of rapid antiviral clearance or clinical benefit with the combination of hydroxychloroquine and azithromycin in patients with severe COVID-19 infection. Med. Mal. Infect..

