# Supplementary material for: Knowledge, Attitude, and Practices of Healthcare Professionals on COVID-19 and Risk Assessment to Prevent the Epidemic Spread: A Multicenter Cross-Sectional Study from Punjab, Pakistan
Source: Int J Environ Res Public Health. 2020 Sep 2;17(17):6395. doi: 10.3390/ijerph17176395 (PMC7503345; doi:10.3390/ijerph17176395)
Supplement: Supplementary file 1 [file ijerph-17-06395-s001.pdf]

Knowledge, Attitude and Practices of Healthcare Professionals on COVID-19 and Risk Assessment to Prevent the Epidemic Spread: A Multicenter Cross-Sectional Study from Punjab Pakistan

Supplementary Material

QUESTIONNAIRE:

Coronavirus disease is proving to be a great threat to the world. The disease is spreading in various parts of the world. Keeping in view the seriousness of the disease, this survey aims to have an idea of the knowledge, attitude, practices and risk assessment of healthcare professionals towards the novel coronavirus.

Participation in the survey is voluntary. You have free will to participate in the study. All the responses and feedback provided by the individuals will be kept confidential and the information of the participants will not be shared with any public organization.

If you decide to participate in the survey, kindly respond to all the questions provided in the survey. Your participation will be highly praised. Thank you for providing us your valuable feedback

SECTION-I (DEMOGRAPHICS)

Age: ____________ years **Gender:** Male Female

Marital Status: Married Unmarried

Highest level of education: Bachelor Master Doctorate

Occupation: Doctor Pharmacist Nurse

Workplace Organization Type: Public Private

Residence: Urban Rural

Table S1. SECTION-II (KNOWLEDGE)

No.	Questions	Yes	No	Don't Know
1	Have you heard about the novel coronavirus and the related terms COVID-19 or 2019-nCoV?			
2	COVID-19 disease is a viral infection.			
3	COVID-19 can be transmitted through close contact with infected people and infected animals.			
4	COVID-19 virus can be transmitted through contaminated food and water.			
5	Fever, sore throat, cough and shortness of breath are possible symptoms of COVID-19 infection.			
6	The novel coronavirus is a similar virus as SARS-CoV and MERS-CoV.			
7	Does the virus survive on surfaces of doors, tables and other objects?			
8	Is the COVID-19 infection the same illness as flu or cold?			

**9**	Is there any laboratory test to confirm the presence of COVID-19 infection?			
**10**	The incubation period of COVID-19 infection is 1-2 weeks.			
**11**	Can COVID-19 infection be caught from a person who presents no symptoms and has recently visited the affected area?			
**12**	Vaccine for COVID-19 virus is now available in the market.			
**13**	Antibiotics are useful for the treatment of COVID-19 infection.			
**14**	People with a compromised immune system and old age people are at more risk of developing the infection.			
**15**	Patients with comorbidities are at more risk of developing the infection.			
**16**	Health care workers and hospitalized patients who are near to infected patients are at more risk of developing the infection.			
**17**	People in crowded places are at increased risk of getting affected by the disease.			
**18**	Patients of COVID-19 infection should be immediately isolated to avoid the transfer of infection to other people.			
**19**	Healthcare professionals are well prepared to cater to the people in case there is a spread of COVID-19 disease.			

Table S2. SECTION-III (ATTITUDES)

No.	Questions	Strongly Disagree	Disagree	Neutral	Agree	Strongly Agree
**1**	The disease can be transmitted by coughing and sneezing.					
**2**	Transmission of COVID-19 infection can be prevented through wearing masks.					
**3**	Transmission of COVID-19 infection can be prevented through washing hands and face regularly with antiseptics and sanitizers					
**4**	Transmission of COVID-19 infection can be prevented through isolation of COVID-19 infected patients.					
**5**	Transmission of COVID-19 infection can be prevented by taking antibiotics.					
**6**	Restricting the travel of COVID-19 infected people to other areas of the world and of people in other areas to affected areas can					

	be beneficial to prevent the spread of the infection.					
**7**	Having a healthy and well-cooked diet can be helpful in reducing the risk of getting the novel corona viral disease.					
**8**	Avoiding touching nose, mouth and eyes can reduce risk of infection.					
**9**	Avoiding touching the surface of doors, furniture or other things can be helpful in preventing the disease.					
**10**	If a vaccine is developed against the novel corona virus, it can significantly reduce the epidemic spread.					
**11**	The available information about COVID-19 disease is sufficient in Pakistani society.					
**12**	The government in our country has all the necessary healthcare facilities and is able to control the epidemic situation.					

Table S3. SECTION-IV (PRACTICES AND RISK ASSESSMENT)

No.	Questions	Always	Mostly	Sometimes	Rarely	Never
**1**	I advise the general public to eat thoroughly cooked food especially meat products.					
**2**	I advise the public to keep themselves warm and hydrated.					
**3**	I advise people to use soap or sanitizer regularly to wash their hands and face.					
**4**	I advise the general public to avoid close contact with people having cough and flu-like symptoms.					
**5**	During interaction with the Covid-19 patient, I wear the necessary personal protective equipment (PPE) such as masks, gloves and					

	gown etc.					
**6**	I perform hand hygiene before and after touching the Covid-19 patients or before and after performing an aseptic procedure.					
**7**	I perform hand hygiene after touching the patient's surroundings like beds, tables, doors etc.					
**8**	I avoid unnecessary close contact and practice social distancing and keep at least 1-meter distance from patients and other healthcare workers.					
